# A High-Fat Diet Increases Gut Microbiota Biodiversity and Energy Expenditure Due to Nutrient Difference

**DOI:** 10.3390/nu12103197

**Published:** 2020-10-20

**Authors:** Botao Wang, Qingmin Kong, Xiu Li, Jianxin Zhao, Hao Zhang, Wei Chen, Gang Wang

**Affiliations:** 1 State Key Laboratory of Food Science and Technology, Jiangnan University, Wuxi 214122, China; jnwangbotao@foxmail.com (B.W.); kongqingmin2@163.com (Q.K.); lixiu@jiangnan.edu.cn (X.L.); zhaojianxin@jiangnan.edu.cn (J.Z.); zhanghao@jiangnan.edu.cn (H.Z.); chenwei66@jiangnan.edu.cn (W.C.); 2 School of Food Science and Technology, Jiangnan University, Wuxi 214122, China; 3 International Joint Research Center for Probiotics & Gut Health, Jiangnan University, Wuxi 214122, China; 4 (Yangzhou) Institute of Food Biotechnology, Jiangnan University, Yangzhou 225004, China; 5 National Engineering Center of Functional Food, Jiangnan University, Wuxi 214122, China; 6 Wuxi Translational Medicine Research Center and Jiangsu Translational Medicine Research Institute Wuxi Branch, Wuxi 214122, China; 7 Beijing Innovation Centre of Food Nutrition and Human Health, Beijing Technology and Business University (BTBU), Beijing 102488, China

**Keywords:** obesity, fiber, high-fat diet, low-fat diet, gut microbiota, SCFAs, glucose homeostasis, energy expenditure

## Abstract

A high-fat diet (HFD) can easily induce obesity and change the gut microbiota and its metabolites. However, studies on the effects of high-fat diets on the host have drawn inconsistent results. In this study, the unexpected results showed that the refined HFD increased gut microbiota diversity and short-chain fatty acids (SCFAs), causing an increase in energy metabolism. Further analysis revealed these changes were caused by the different fiber content in these two diets. Male C57BL/6J mice (4–5 weeks old) were fed either HFD or refined low-fat diet (LFD) for 14 weeks. The metabolic rates, thermogenesis, gut microbiome, and intestinal SCFAs were tested. The HFD triggered obesity and disturbed glucose homeostasis. Mice fed HFD ingested more fiber than mice fed LFD (*p* < 0.0001), causing higher intestinal SCFA concentrations related to the increased abundances of specific bacteria in the HFD group. Also, the HFD increased metabolic heat and up-regulated thermogenesis genes uncoupling protein 1(*Ucp-1*), peroxisome proliferator-activated receptor-γ coactivator-1α (*Pgc-1α*) expression in the brown adipose tissue (BAT). It was revealed by 16S rRNA gene sequencing that the HFD increased gut microbial diversity, which enriched Desulfovibrionaceae, *Rikenellaceae RC9 gut group*, and *Mucispirillum*, meanwhile, reduced the abundance of *Lactobacillus*, *Bifidobacterium*, *Akkermansia*, *Faecalibaculum*, and *Blautia*. The predicted metabolic pathways indicated HFD increased the gene expression of non-absorbed carbohydrate metabolism pathways, as well as the risks of colonization of intestinal pathogens and inflammation. In conclusion, the HFD was obesogenic in male C57BL/6J mice, and increased fiber intake from the HFD drove an increase in gut microbiota diversity, SCFAs, and energy expenditure. Meanwhile, the differences in specific nutrient intake can dissociate broad changes in energy expenditure, gut microbiota, and its metabolites from obesity, raising doubts in the previous studies. Therefore, it is necessary to consider whether differences in specific nutrient intake will interfere with the results of the experiments.

## 1. Introduction

Obesity is a non-communicable disease characterized by excessive fat accumulation, which can trigger serious health problems [1,2]. Obesity has been the subject of intensive research as it has become prevalent worldwide and is considered a severe threat to public health. What is worse, obesity has become apparent in increasingly younger patients, and the prevalence of obesity-associated morbidity in children and young adults has rapidly risen.

Globally, scientists have gradually reached a consensus in studying obesity: a disequilibrium between energy intake and expenditure results in weight gain and eventually obesity. The proportion of patients with obesity is significantly higher in Western countries than in other parts of the world. Although researchers have proposed for centuries that diets might contribute to obesity, systematic studies of the diets have only become a subject of interest in recent decades [3]. The Western diet is characterized by a high intake of meat, fat, and deep-processed, low-fiber carbohydrates [4]. Between 1998 and 2009, the mean daily dietary fiber intake in the USA was significantly lower than the recommended adequate intake, and individuals with obesity ingested significantly less dietary fiber than healthy individuals [5]. Increasing dietary fiber intake has been reported to assist in preventing obesity by increasing the intestinal production of short-chain fatty acids (SCFAs) [6,7,8].

A healthy diet not only offers essential nutrition to the host but also promotes a healthy gut microbiome. Diets affect the gut microbial composition, a factor that is closely linked with obesity [9,10,11]. Either high-fat or high-sugar diets have been proved to result in overweight and obesity, in addition to altering the gut microbiota composition [12,13,14], such as by reducing microbial diversity and reducing the abundance of *Bifidobacterium* and *Akkermansia* [15,16]. Immigrants from non-Western countries to the USA change their dietary habits, causing significant perturbations to their gut microbial composition, reducing biodiversity and fiber-degrading microbes, which increases their risk of obesity [17].

In the animal studies on obesity, a low-fat diet or a standard chow diet is usually selected as the control group. There is little difference in the nutrient ratio or energy density between the LFD and the standard chow diet, but there is a big difference in the composition of some nutrients. Therefore, the selection of control diets will lead to different or even opposite conclusions. This article compares the effects of a high-fat diet (HFD) and a low-fat diet (LFD) on obesity and finds that the difference in nutrient intake from the diets affects the physiological function, energy metabolism, gut microbiota, and its metabolites of mice.

## 2. Materials and Methods

### 2.1. Animals and Experimental Design

Only male C57BL/6J (4- to 5-week-old) mice were used in this study, purchasing from Gempharmatech Co., Ltd., Nanjing, China. The mice were housed in SPF-grade laboratory animal facility with a temperature (21 °C ± 2 °C) and humidity (55% ± 10%) controlled barrier environment under a standard 12-h:12-h light: dark cycle with free access to water and food. The project license was approved by the Ethics Committee of Experimental Animals at Jiangnan University (qualified number: JN.No20181230c0880715 (284)). The mice were adapted for one week, then divided into two groups (*n* = 8 per group) dependent on two diets: refined HFD (TP 23300, TROPHIC Animal Feed High-tech Co., Ltd., Nantong, China), supplying energy as 20% carbohydrate (7% sucrose calories), 20% protein, 60% fat with total 5.1 kcal/g; and refined LFD (TP23302, TROPHIC Animal Feed High-tech Co., Ltd., Nantong, China), supplying energy as 70% carbohydrate (7% sucrose calories), 20% protein, 10% fat with total 3.8 kcal/g, allocating for the HFD and LFD groups. The composition of the two diets is shown in Table 1. The HFD and LFD were weighed and replaced every two days to keep them fresh. The weight of the mice was recorded twice every week. After feeding HFD or LFD for 14 weeks, the mice were anesthetized through intraperitoneal injection with 1% pentobarbital sodium solution at a dose of 40 mg/kg, and an intracardiac puncture was immediately performed to collect fresh blood samples. The mice were sacrificed by cervical dislocation. The weight of abdominal white adipose tissue, interscapular brown adipose tissue (BAT), liver, and spleen were measured. These tissues and all intestinal tracts containing contents were put into liquid nitrogen.

### 2.2. Oral Glucose Tolerance Test

In all mice from each diet, an oral glucose tolerance test (OGTT) was performed at the 14th week. After a fasting period of 12 h, the test was conducted by intragastric administration with glucose at 2 mg/g body weight in mice. Blood was collected from the tail vein before intragastric administration and at 30, 60, 90, 120 min. The fresh blood samples were directly tested by Accu-Chek Active test strips (Roche Diabetes Care, Mannheim, Germany). Areas under the curve (AUC) for OGTTs were calculated by Graphpad Prism 6 (GraphPad Software, San Diego, CA, USA).

### 2.3. Biochemical Measurements

Blood samples clotted naturally at room temperature for 2 h, then were centrifuged at 1000× *g* for 15 min. Supernatants were collected to perform serum related indicators. The concentrations of serum alanine aminotransferase (ALT), aspartate aminotransferase (AST), total cholesterol (TC), triacylglycerol (TG), high-density lipoprotein cholesterol (HDL-C), and low-density lipoprotein cholesterol (LDL-C) were analyzed by automatic biochemistry analyzer (BS-480, Mindray, Shenzhen, China).

### 2.4. Metabolic Rates Analysis

Metabolic rates were performed in a Comprehensive Lab Animal Monitoring System (Columbus Instruments, Columbus, OH, USA). The data collection and analysis were reported in the study of Sungsoon et al. [18].

### 2.5. Gene Expression Analysis

Total RNA was isolated from frozen mouse BAT and liver (stored at −80 °C) using TRIzol reagent (15596026, Invitrogen, Life Technologies Corporation, Carlsbad, CA, USA) according to the user guide. The cDNA was synthesized using HiFiScript gDNA Removal RT MasterMix (CW2020, Cowinbioscience Co., Ltd., Beijing, China). Quantitative real-time polymerase chain reaction (qPCR) was performed on the BioRad-CFX384 Touch thermocycler using ChamQ Universal SYBR qPCR Master Mix (Q711, Vazyme Biotech Co., Ltd., Nanjing, China). The primers were listed in Table 2. The PCR program and data analysis were reported in a previous study [19].

### 2.6. Gut Microbiota Sequencing

Total DNA was extracted from intestinal contents (cecum and colon) by Fast DNA Stool Kit (M.P. Biomedicals, Irvine, CA, USA), and used for PCR amplification of the V3-V4 regions of 16S rRNA gene by barcoded primers 341F and 806R. The PCR amplification products were purified by nucleic acid gel electrophoresis, and the amplicons on the gels were purified via a PCR purification kit (TIANgel Mini Purification Kit, TIANGEN, Beijing, China). The pooled equimolar mix of purified amplicons was performed on an Illumina MiSeq PE300 platform (Illumina, San Diego, CA, USA). The composition and function of the gut microbiome were assessed using Quantitative Insights Into Microbial Ecology2 (QIIME2) [20] and Tax4Fun2 [21].

### 2.7. Detection of Short-Chain Fatty Acids (SCFAs) from Intestinal Contents

Fresh intestinal contents were dried via vacuum freeze drier, and the dry weight was measured. SCFAs were extracted using diethyl ether according to reported protocols [22,23] and some modifications. The extracts were measured by a gas chromatography-mass spectrometer (GC-MS, QP2010 Ultra, SHIMADZU, Kyoto, Japan) on the Rtx-Wax capillary column with Helium as a carrier gas at 0.89 mL/min. The inlet temperature was 240 °C, and the initial oven temperature was 100 °C. After injection, the oven temperature rose to 140 °C at a rate of 7.50 °C/min, then rose to 200 °C at 60 °C/min and kept for three minutes. The mass spectrometer parameters were set as follows: ion source temperature: 220 °C; Interface temperature: 250 °C; Solvent delay: 2.5 min; m/z scan range: 2 to 100.

### 2.8. Statistical Analysis

Data were shown as means ± standard deviation (S.D.), and the differences between variants were analyzed using unpaired two-tailed Student’s *t*-test unless otherwise stated. All of the statistical analyses were carried on GraphPad Prism 6 (version 6.01, GraphPad Software Inc., San Diego, CA, USA), SPSS 19.0 (SPSS Inc., Chicago, IL, USA), and statistical analysis of taxonomic and functional profiles (STAMP) [24]. Principal component analysis (PCA), orthogonal partial least square-discriminate analysis (OPLS-DA), and variable importance for the projection (VIP) for the predictive components (VIP predictive) were carried out on SIMICA 14 (Umetrics, Umeå, Sweden). Gut microbiota analysis was performed on QIIME 2, Tax4Fun2, and R (version 3.6.1) software. Linear discriminant analysis (LDA) effect size (LEfSe) was according to previous procedure [25].

## 3. Results

### 3.1. High-Fat Diet (HFD) Triggered Obesity and Disrupted Glucose Homeostasis

**Figure 1:**
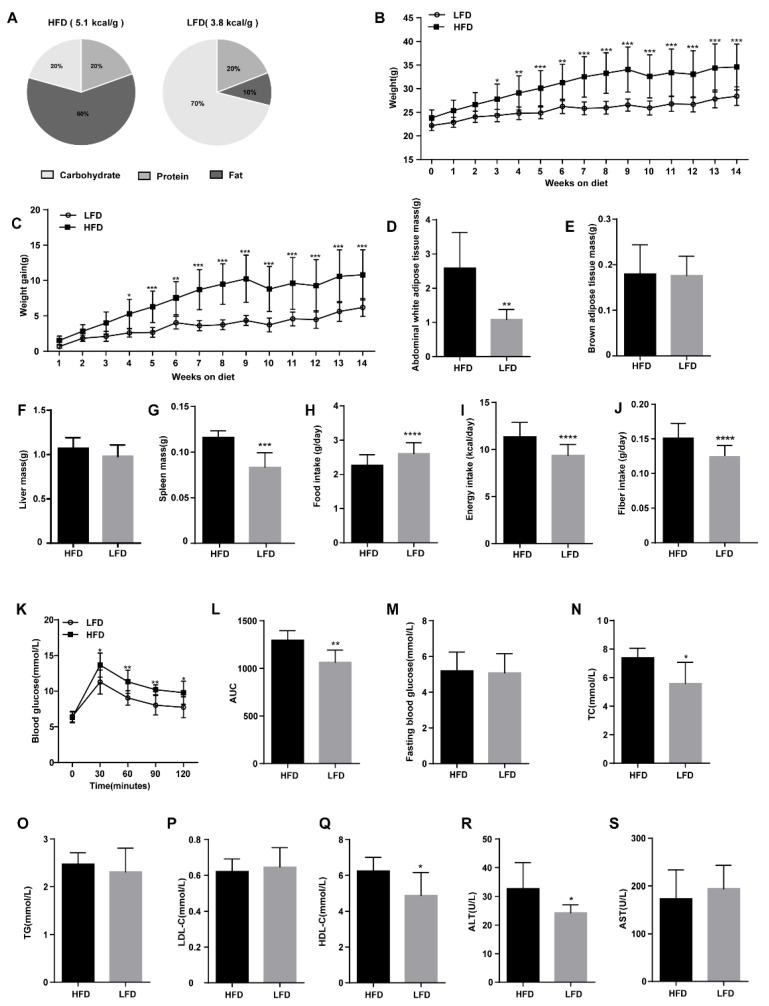
Effects of high-fat diet (HFD) and low-fat diet (LFD) on mouse physiology and glucose control. (**A**) Composition of dietary nutrition. (**B**) Weekly body weight of mice fed an HFD or LFD. (**C**) Weekly body weight gain by mice fed an HFD or LFD. (**D**–**G**) Weight of different tissues at 14th week. (**H**) Mean daily food intake. (**I**) Mean daily energy intake. (**J**) Fiber intake. (**K**) Blood glucose concentrations of oral glucose tolerance test (OGTT). (**L**) The area under the curve (AUC) of blood glucose concentrations. (**M**) Fasting blood glucose concentrations. (**N**–**S**) Concentrations of blood biochemical indices, namely total cholesterol (TC), triacylglycerol (TG), low-density lipoprotein cholesterol (LDL-C), high-density lipoprotein cholesterol (HDL-C), alanine aminotransferase (ALT), and aspartate aminotransferase (AST). Mean values ± standard deviation (S.D.) are plotted. Asterisks indicate significant differences (unpaired two-tailed Student’s *t*-test, * *p* < 0.05, ** *p* < 0.01, *** *p* < 0.001, **** *p* < 0.0001, *n* = 8 mice per group).

Both the HFD and LFD were processed from the same purified ingredients with different compositions of specific nutrients (Figure 1A). After three weeks on the diets, the weight of the mice in the LFD group was significantly lower than that in the HFD group (Figure 1B). By the fourth week, the weight gain of the HFD group was significantly different from the LFD group (Figure 1C). The weight gain in mice fed the HFD was higher than that in mice fed the LFD (*p* = 0.0043, 10.80 ± 3.55 g vs. 6.19 ± 1.27 g). After 14 weeks, the mice fed the HFD had an increased mass of abdominal white adipose tissue (Figure 1D), but there was no difference in the mass of brown fat between the HFD and the LFD group (Figure 1E). There was no significant difference in the liver mass between the mice fed the HFD and LFD, but the splenic mass of the HFD and LFD groups differed significantly (Figure 1F,G). Interestingly, the LFD mice consumed more food daily (Figure 1H) but gained less weight as the HFD was more energy-dense than the LFD, and thus the daily energy intake of mice fed the HFD was much higher than the LFD group (*p* < 0.0001; 11.33 ± 0.19 kcal/day vs. 9.38 ± 0.14 kcal/day, Figure 1I). Moreover, the mice fed HFD ingested a greater amount of fiber (cellulose) compared to the mice fed LFD (*p* < 0.0001, 0.152 ± 0.021 g/day vs. 0.125 ± 0.015 g/day, Figure 1J).

After 14 weeks, the fasting blood glucose levels of HFD and LFD mice showed no significant difference (Figure 1M). Nevertheless, an OGTT revealed that blood glucose of the HFD group was rapidly elevated compared to the LFD group after 30 min of oral glucose, with the HFD group maintaining a higher blood glucose concentration for the following 90 min (Figure 1K). In contrast to the LFD mice, the AUC for the HFD group mice was increased significantly (Figure 1L). Serum TC, HDL-C, and ALT levels were significantly higher in the HFD fed mice than those in the LFD fed mice; however, no difference was found in serum TG, LDL-C, and AST levels between these groups. (Figure 1N–S).

### 3.2. HFD Enhanced Energy Expenditure and Lipid Metabolism

**Figure 2:**
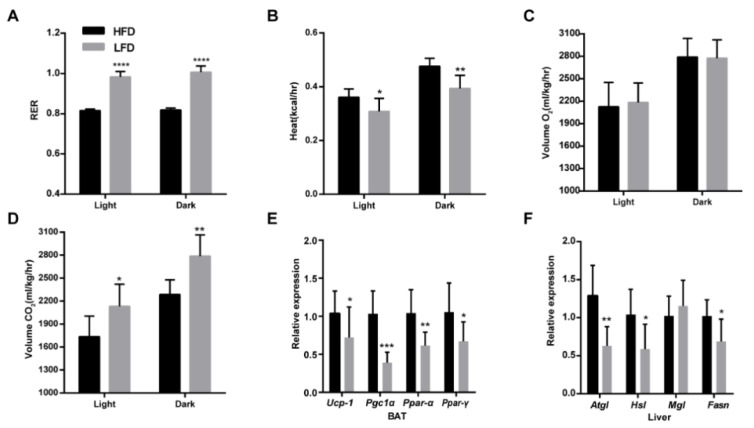
HFD enhanced energy expenditure and lipid metabolism. (**A**) The relative rates of carbohydrate versus fat oxidation (R.Q.) in the light and dark. (**B**) Metabolic heat in the light and dark. (**C**) Consumption of O_2_ in the light and dark. (**D**) Production of CO_2_ in the light and dark. (**E**) Relative expression of thermogenesis genes uncoupling protein 1(*Ucp-1*), peroxisome proliferator-activated receptor-γ coactivator-1α (*Pgc-1α*), peroxisome proliferator-activated receptor-alpha (*Ppar-α*), and peroxisome proliferator-activated receptor-gamma (*Ppar-γ*) in the BAT. (**F**) Relative expression of lipid metabolism genes patatin-like phospholipase domain-containing protein 2 (*Atgl*), hormone-sensitive lipase (*Hsl*), acylglycerol lipase (*Mgl*), and fatty acid synthase (*Fasn*) in the liver. Mean values ± S.D. are plotted. Asterisks indicate significant differences (unpaired two-tailed Student’s *t*-test, * *p* < 0.05, ** *p* < 0.01, *** *p* < 0.001, **** *p* < 0.0001, *n* = 7–8 mice per group).

The average daily energy intake of mice in the HFD group was 1.95 kcal more than that in the LFD group. If the increased energy was stored as fat, the mice fed HFD should gain more weight. Therefore, we speculate that the daily energy metabolism of the HFD mice may be higher than that of the LFD mice. Based on this hypothesis, we tested the metabolic rates between the HFD and LFD group. The results showed that the relative rates of carbohydrate versus fat oxidation (RQ) in the HFD group were significantly lower than those in the LFD group whether in the light or the dark (Figure 2A). Meanwhile, the heat production of HFD group mice was higher than that of LFD group mice, especially in the dark (*p* = 0.0054, 0.4760 ± 0.02968 kcal/h vs. 0.3937 ± 0.04877 kcal/h, Figure 2B). There was no difference in the O_2_ consumption between the HFD group and the LFD group (Figure 2C), but the mice fed LFD produced more CO_2_ compared to the mice fed HFD, which was more evident in the dark (*p* = 0.0046, 2286 ±192.9 mL/kg/h vs. 2786 ± 277.3 mL/kg/h, Figure 2D). Based on the differences in the metabolic rates, we further tested the genes related to thermogenesis in the BAT and found that the expressions of *Ucp-1*, *Pgc1α*, *Ppar-α*, and *Ppar-γ* genes in the HFD group were significantly higher than those in the LFD group, which was consistent with the metabolic rate of heat (Figure 2E). At the same time, HFD also caused significant up-regulation of the lipase genes *Atgl*, *Hsl*, and fatty acid synthase gene *Fasn* in the liver of mice, indicating that the lipid metabolism activity of mice fed HFD was more vigorous than the mice fed LFD (Figure 2F).

### 3.3. HFD Increased Biodiversity and Influenced Gut Microbial Composition in the Cecum

**Figure 3:**
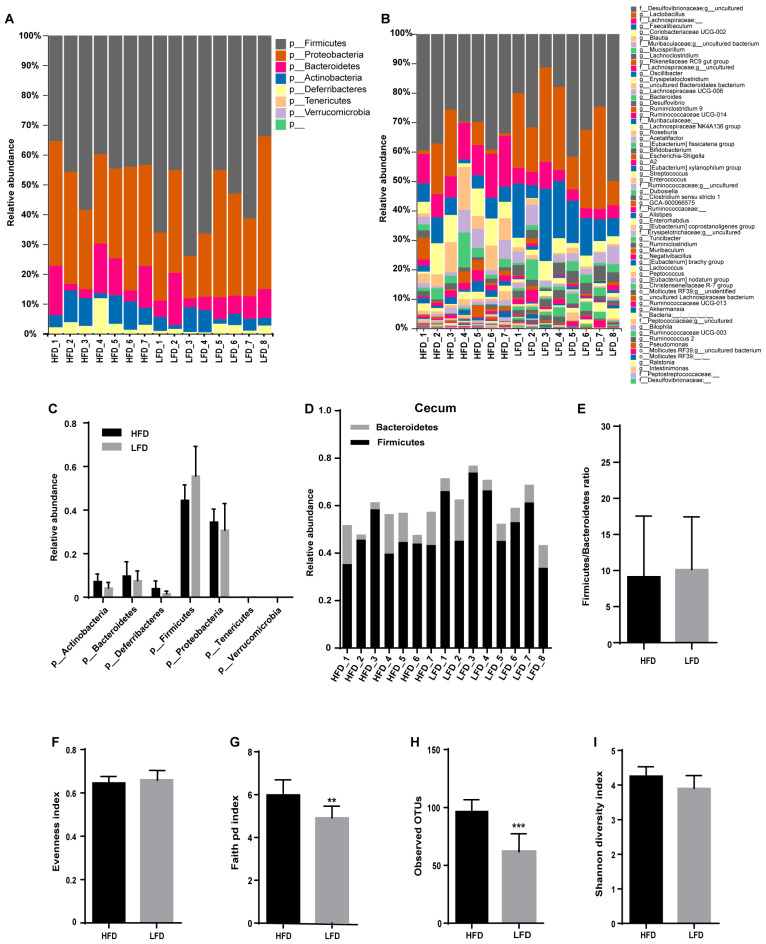
Effect of diets on cecal microbiota composition. (**A**,**B**) Microbiota composition by phylum and genus in the cecum of the HFD and the LFD mice. (**C**) The relative abundance of gut microbiota by phylum. (**D**) The relative abundance of Bacteroidetes and Firmicutes. (**E**) Firmicutes:Bacteroidetes ratio for the HFD and the LFD groups. (**F**–**I**) Alpha-diversity indices of gut microbiota for the HFD and the LFD groups. Mean values ± S.D. are plotted. Asterisks indicate significant differences (unpaired two-tailed Student’s *t*-test, ** *p* < 0.01, *** *p* < 0.001, *n* = 7–8 mice per group).

Different diets shape the different composition of gut microbiota. The gut microbiomes of mice fed an HFD or an LFD were mainly composed of Firmicutes, Bacteroidetes, Proteobacteria, and Actinobacteria phyla (Figure 3A,C). There was no significant difference in either the abundance of Firmicutes or the Firmicutes:Bacteroidetes ratio between HFD and LFD groups (Figure 3C–E). Observed operational taxonomic unit (OTU) analyses revealed that 96 OTUs in the HFD group and 61 OTUs in the LFD group were identified (Figure 3B,H) and Faith’s Phylogenetic Diversity (P.D.) was much higher in the HFD group than that in the LFD group (Figure 3G).

**Figure 4:**
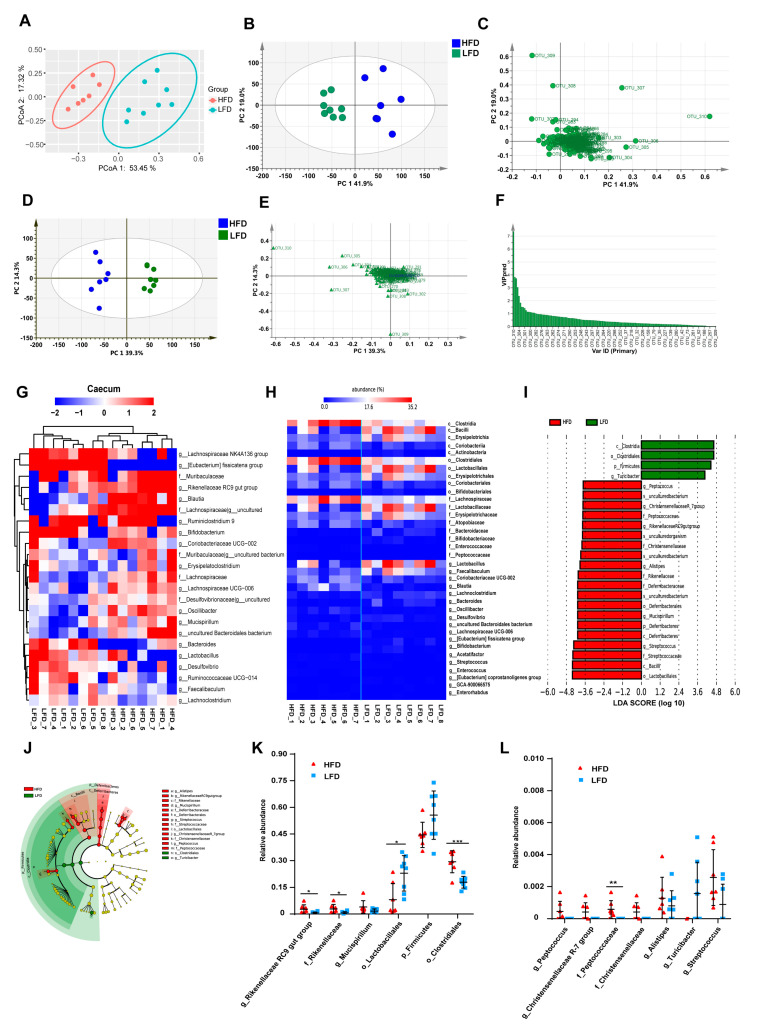
Different diets resulted in different gut microbial clusters and different dominant bacteria in the cecum. (**A**) Principal coordinate analysis (PCoA) of gut microbiota for the HFD and the LFD groups (PCoA 1 = 53.5%, PCoA 2 = 17.3%). (**B**) Principal component analysis (PCA) score scatter plots for gut microbiota for the HFD and the LFD (PC 1 = 41.9%, PC 2 = 19.0%). (**C**) PCA loading scatter plot of gut microbiota for the HFD and the LFD groups. (**D**) The orthogonal partial least square-discriminate analysis (OPLS-DA) score scatter-plot of gut microbiota for the HFD and the LFD groups (PC 1 = 39.3%, PC 2 = 14.3%). (**E**) The OPLS-DA loading scatter plot of gut microbiota for the HFD and the LFD groups. (**F**) Values of variable importance for the projection (VIP) for the predictive components (VIP predictive) of gut microbiota. (**G**) Heatmap of the proportion of the 23 operational taxonomic units (OTUs) determined as dominant bacteria, with rows clustered by microbiota similarity according to the Euclidean distance, and columns clustered by operational taxonomic units (OTUs) that occur more often together. (**H**) Heatmap of the statistical analysis of taxonomic and functional profiles (STAMP) showed significant differences in the microbiota of the HFD and the LFD groups. (**I**) Linear discriminant analysis (LDA) scores of gut microbiota for the HFD and the LFD groups. (**J**) Cladograms representing the LDA effect size (LEfSe) results for the HFD and the LFD groups. (**K**,**L**) Dot plots representing the proportional abundance of OTUs from LEfSe results. Mean values ± S.D. are plotted. Asterisks indicate significant differences (unpaired two-tailed Student’s *t*-test, * *p* < 0.05, ** *p* < 0.01, *** *p* < 0.001, *n* = 7–8 mice per group).

Principal coordinate analysis (PCoA) showed that the samples within each group were clustered into their respective groups (PCoA 1 = 53.5%, PCoA 2 = 17.3%, Figure 4A). Score and loading scatter-plots based on principal component analysis (PCA) showed similar results to the PCoA, with samples from mice in each group being distributed into two clusters (PC 1 = 41.9%, PC 2 = 19.0%, Figure 4B,C). The score and loading scatter-plots of OPLS-DA also showed similar results (Figure 4D,E). The values of the VIP for the predictive components (VIP predictive) were greater than 1, indicating that these OTUs were probably differentially abundant in the microbiomes (Figure 4F), including *Lactobacillus*, two uncultured bacteria in Desulfovibrionaceae, seven OTUs in Lachnospiraceae (*Eubacterium fissicatena group*, *Blautia*, *Lachnospiraceae UCG-006*, and four unclassified OTUs), *Oscillibacter*, *Erysipelatoclostridium*, *Mucispirillum*, *Rikenellaceae RC9 gut group*, *Coriobacteriaceae UCG-002* and two uncultured bacteria in Muribaculaceae.

Based on the results of relative abundances, 23 OTUs were selected as the dominant bacteria that accounted for more than 90% of the total microbial abundance (Figure 4G). Figure 4H showed significant differences in the gut microbiota composition between the HFD and LFD groups at the levels of class, order, family, and genus. The heatmap indicated that the samples were clustered into two parts, with six samples from the LFD group classified into one cluster, and the remainder clustered separately (Figure 4H). The relative abundances of *Lactobacillus*, *Faecalibaculum*, *Lachnoclostridium*, *Bacteroides*, *Desulfovibrio*, *Eubacterium fissicatena group*, and *Bifidobacterium* were significantly higher in the LFD group than that in the HFD group. In contrast, Lachnospiraceae, *Blautia*, *Rikenellaceae RC9 gut group*, *Oscillibacter*, an uncultured Bacteroidales bacterium, *Lachnospiraceae UCG-006* in the HFD group were more abundant. LEfSe results indicated that the taxa feature that best characterize the differences between the HFD and LFD groups were mainly those of the Rikenellaceae, Deferribacteraceae, Streptococcaceae, Christensenellaceae, and Peptococcaceae families (Figure 4I,J). The relative abundances of differentially abundant bacteria identified by LEfSe revealed the abundances of *Rikenellaceae RC9 gut group*, Rikenellaceae, Clostridiales, and Peptococcaceae were significantly higher in the HFD group, while Lactobacillae were more abundant in the LFD group (Figure 4K,L).

### 3.4. Low-Fat Diet (LFD) Reduced Colonic Gut Microbial Biodiversity But Increased the Retention of Beneficial Bacteria

**Figure 5:**
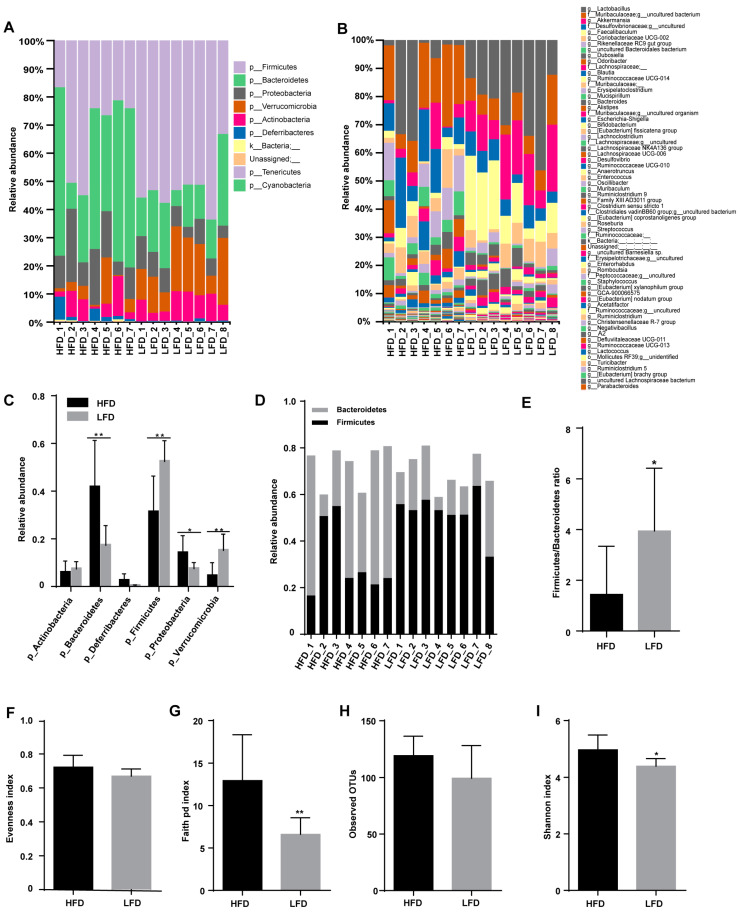
Effect of diets on colonic microbial composition. (**A**,**B**) Microbial composition by phylum and genus within the colon of mice fed an HFD or an LFD. (**C**) Relative abundances of gut microbiota by phylum. (**D**) The relative abundance of Bacteroidetes and Firmicutes. (**E**) Firmicutes:Bacteroidetes ratio for the HFD and the LFD. (**F**–**I**) Alpha diversity indices of gut microbiota for the HFD and the LFD. Mean values ± S.D. are plotted. Asterisks indicate significant differences (unpaired two-tailed Student’s *t*-test, * *p* < 0.05, ** *p* < 0.01, *n* = 7–8 mice per group).

The refined diets not only changed gut microbiota in the cecum but also affected the microbial composition in the colon. The relative abundance histograms at the phylum and genus levels revealed that the composition of gut microbiota within colonic contents was various between HFD and LFD groups (Figure 5A,B). The gut microbiota present at the phylum level comprised Firmicutes, Bacteroidetes, Proteobacteria, Verrucomicrobia, Actinobacteria, and Deferribacteres, a composition that was different from that seen in the cecum. The relative abundances of Bacteroidetes and Proteobacteria were higher in the HFD group, while the relative abundances of Firmicutes and Verrucomicrobia in the HFD group were lower than that in the LFD group (Figure 5C). The relative abundance histogram and bar plot indicated that the Firmicutes:Bacteroidetes ratio was significantly higher in the LFD group than in the HFD group (Figure 5D,E). Analysis with alpha-diversity indices, namely Faith’s P.D. index and Shannon’s index, showed higher alpha-diversity in the HFD group, indicating more phylogenetic diversity within the HFD group than the LFD group (Figure 5F–I).

**Figure 6:**
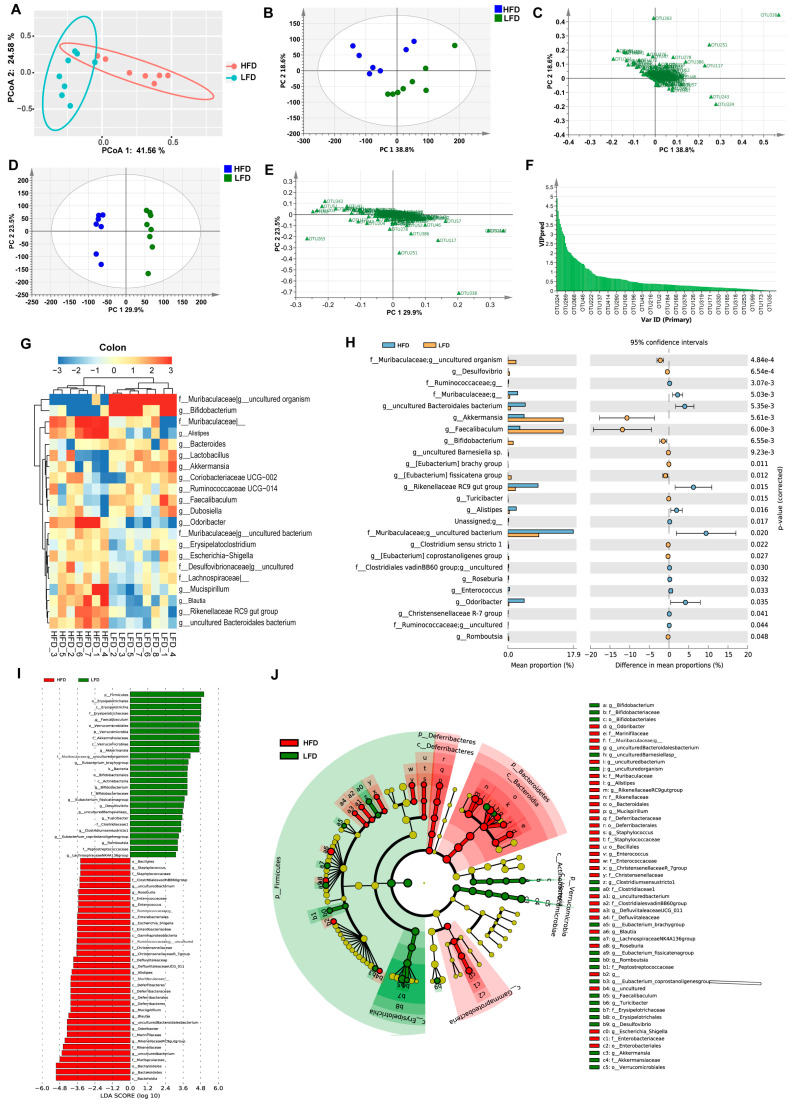
Different diets result in different gut microbiota clusters and different dominant bacteria in the colon. (**A**) PCoA of gut microbiota for the HFD and the LFD (PCoA 1 = 41.56%, PCoA 2 = 24.58%). (**B**) PCA score scatter plot of gut microbiota for the HFD and the LFD (PC 1 = 38.8%, PC 2 = 18.6%). (**C**) PCA loading scatter plot of gut microbiota for the HFD and the LFD. (**D**) The OPLS-DA score scatter plot of gut microbiota for the HFD and the LFD (PC 1 = 29.9%, PC 2 = 23.5%). (**E**) The OPLS-DA loading scatter plots of gut microbiota for the HFD and the LFD. (**F**) VIP predictive of gut microbiota. (**G**) Heatmap of the proportion of the 21 OTUs determined as dominant bacteria, with rows clustered by microbiota similarity using the Euclidean distance, and columns clustered by OTUs that occur more often together. (**H**) The extended error bar plot showed significantly different microbial communities existing between the HFD and the LFD groups. (**I**) LDA scores of gut microbiota for the HFD and the LFD group. (**J**) Cladograms were representing the LEfSe results for the HFD and the LFD groups. (*n* = 7–8 mice per group).

PCoA plots illustrating samples of the HFD and LFD groups show that these groups were clustered into two parts with a large distance between them (Figure 6A), and similar results were observed in PCA results (Figure 6B,C), as well as the results of OPLS-DA (Figure 6D,E). The 33 OTUs were filtered for those with a VIP predictive score >1 (Figure 6F), comprising *Akkermansia*, *Faecalibaculum*, uncultured Bacteroidales bacterium, *Odoribacter*, *Rikenellaceae RC9 gut group*, *Lactobacillus*, *Blautia*, *Mucispirillum*, *Bacteroides*, *Alistipes*, *Bifidobacterium*, *Escherichia-Shigella*, *Eubacterium fissicatena group*, *Alistipes*, *Ruminococcaceae UCG-014*, *Enterococcus*, an uncultured Desulfovibrionaceae bacterium and an uncultured Muribaculaceae bacterium. Twenty-one OTUs were selected as the dominant bacteria, accounting for more than 90% of the abundance (Figure 6G). The heatmap of these dominant bacteria indicated that the samples were clustered into two groups with an obvious difference in the relative abundances of the bacteria in these two groups. STAMP revealed that 25 OTUs were significantly different between the HFD and LFD groups, namely nine OTUs of dominant bacteria: *Akkermansia*, *Alistipes*, *Bifidobacterium*, *Faecalibaculum*, *Odoribacter*, *Rikenellaceae RC9 gut group*, uncultured Bacteroidales bacterium, and Muribaculaceae uncultured bacteria (Figure 6H). LEfSe results indicated that different taxa were assigned to the phyla Verrucomicrobia, Firmicutes, Deferribacteres, and Bacteroidetes (Figure 6I,J). There were 28 OTUs with significantly discriminative features between the HFD and LFD groups, with 11 OTUs being dominant bacteria, namely *Akkermansia*, *Alistipes*, *Bifidobacterium*, *Blautia*, *Faecalibaculum*, *Mucispirillum*, *Odoribacter*, *Rikenellaceae RC9 gut group*, uncultured Bacteroidales bacterium, and Muribaculaceae uncultured bacteria.

### 3.5. Predicted Metabolic Pathways

**Figure 7:**
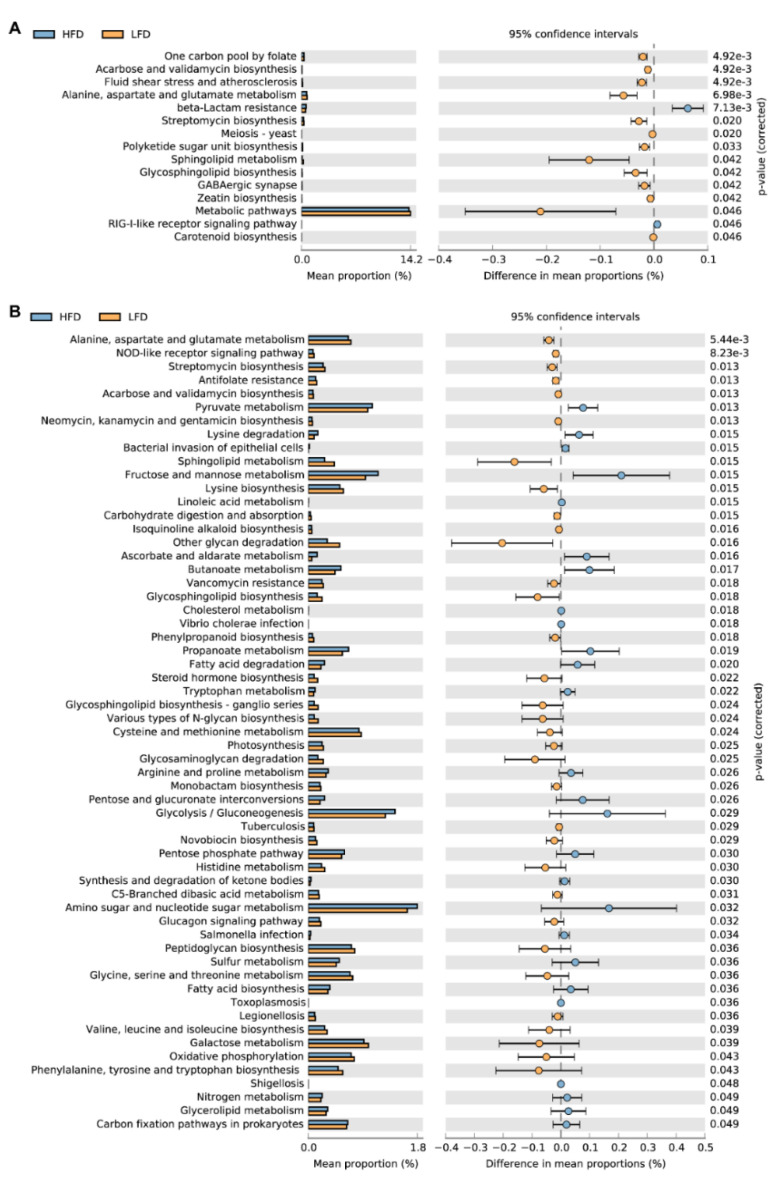
The predicted functions of the gut microbiome by Tax4Fun evaluated the differences in the Kyoto Encyclopedia of Genes and Genomes (KEGG) pathway. (**A**) The differences in the KEGG pathways between mice fed HFD and LFD in the cecum. (**B**) There were 217 pathways different between mice fed HFD and LFD. 59 pathways with a high mean proportion were selected for analysis. The extended error bar shows significant differences (two-sided Welch’s *t*-test with storey false discovery rate (FDR) for multiple test correction).

Tax4fun2 was used to predict the function of the 16S rRNA gene sequences to evaluate the differences in the Kyoto Encyclopedia of Genes and Genomes (KEGG) pathway between HFD and LFD fed mice. In total, there were differences in 15 KEGG pathways between the HFD group and the LFD group in the cecum (Figure 7A). The beta-Lactam resistance and RIG-I-like receptor signaling pathways were significantly increased in the HFD group. In other metabolic pathways, mice in the LFD group were higher than those in the HFD group.

The predicted functions of colonic gut microbiota showed that 217 pathways were different between mice fed HFD and LFD. Therefore, we selected 59 pathways with a high mean proportion for further analysis (Figure 7B). In the carbohydrate metabolism pathway, the functional compositions of pyruvate metabolism, propanoate metabolism, and butanoate metabolism pathways were significantly increased in the HFD group. In the biosynthesis of other secondary metabolites pathway, especially the pathways related to antibiotic synthesis, the functional compositions of the LFD group were significantly higher than the HFD group. Also, in the pathways of bacterial infectious diseases, the functional compositions of the HFD group were significantly higher than those of the LFD group. The predicted functional genes of the LFD group were significantly higher than those in the HFD group in 7 pathways of amino acid metabolism. The functional compositions of arginine and proline metabolism, lysine degradation, and tryptophan metabolism were increased in the HFD group.

### 3.6. HFD Promoted the Production of Intestinal SCFAs Dependent on Specific Bacteria Colonization

**Figure 8:**
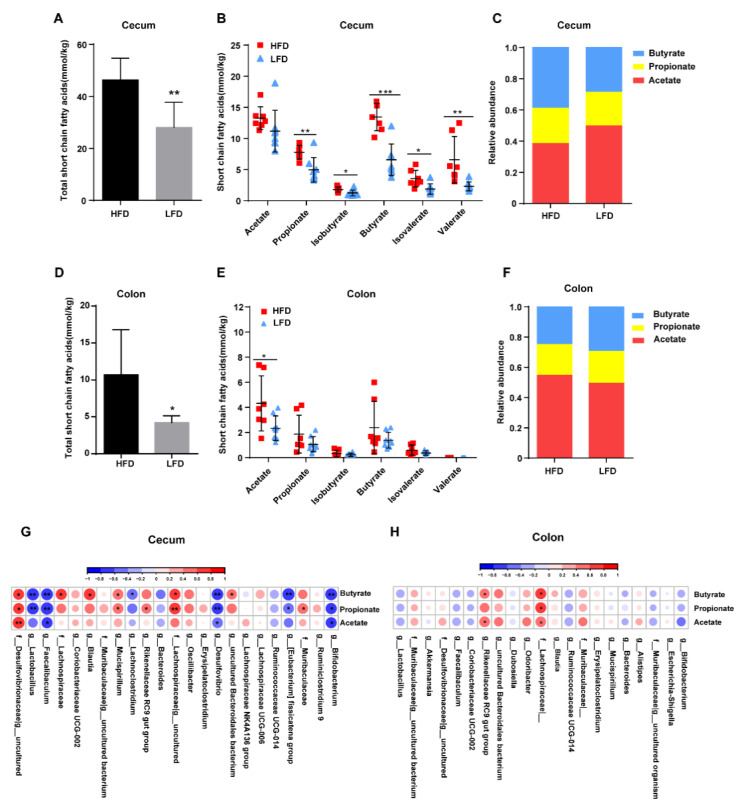
Effects of diets on intestinal short-chain fatty acid (SCFA) production. (**A**) Total SCFAs concentrations in the cecum. (**B**) Cecal concentrations of acetate, propionate, isobutyrate, butyrate, isovalerate, and valerate. (**C**) Relative proportions of acetate, propionate, and butyrate in the cecum. (**D**) Total SCFAs in the colon. (**E**) Colonic concentrations of acetate, propionate, isobutyrate, butyrate, isovalerate, and valerate. (**F**) Relative proportions of acetate, propionate, and butyrate in the colon. (**G**) Correlation of cecal dominant bacteria with SCFA concentrations. (**H**) Correlation of colonic dominant bacteria with SCFA concentrations. Mean values ± S.D. are plotted. Asterisks indicate significant differences were analyzed using unpaired two-tailed Student’s *t*-test (* *p* < 0.05, ** *p* < 0.01, *** *p* < 0.001, *n* = 7–8 mice per group), and Pearson correlation analysis was performed in (**G**,**H**).

As the HFD was found to alter the microbiota composition in both the cecum and colon of mice, the concentrations of SCFAs were measured in the intestinal contents of the HFD and LFD groups. It was found that the concentrations of the SCFAs were significantly different between the two groups. Interestingly, a greater level of SCFAs from the cecum was detected in the HFD group than in the LFD group, such as propionate, isobutyrate, butyrate, isovalerate, and valerate (Figure 8A,B). The relative proportions of acetate, propionate, and butyrate indicated that the ratio of acetate to butyrate in the cecum was different in the two groups (*p* = 0.0002, HFD vs. LFD: 0.9994 ± 0.1428 vs. 1.770 ± 0.3525, Figure 8C). Overall, the total concentrations of SCFAs in the colon were significantly lower than in the cecum (Figure 8A,D). The concentration of total SCFAs in the colon was higher in the HFD group compared to the LFD group (Figure 8D), as well as the concentration of acetate (Figure 8E). Acetate was present in the highest concentrations of all the SCFAs measured in both groups (Figure 8F).

Correlation analysis of the dominant bacteria and the SCFAs detected in the cecum (Figure 8G) showed that an uncultured Desulfovibrionaceae bacterium was positively correlated with the concentration of acetate, propionate, and butyrate, while *Faecalibaculum*, *Desulfovibrio*, and *Bifidobacterium* were negatively correlated with acetate, propionate and butyrate production. *Mucispirillum*, *Rikenellaceae RC9 gut group*, an uncultured Lachnospiraceae bacterium, and Muribaculaceae were positively correlated with propionate, whereas *Lactobacillus* and *Eubacterium fissicatena group* showed negative correlations with propionate. *Lachnospiraceae*, *Blautia*, *Mucispirillum*, an uncultured Lachnospiraceae bacterium, and Muribaculaceae uncultured Bacteroidales bacterium were positively correlated with butyrate, while *Lactobacillus*, *Lachnoclostridium*, and *Eubacterium fissicatena group* showed negative correlations. In the colon, *Rikenellaceae RC9 gut group* was positively correlated with acetate and butyrate, and Lachnospiraceae was positively correlated with the concentration of acetate, propionate, and butyrate (Figure 8H). Interestingly, the dominant bacteria that were positively correlated with SCFAs showed a negative correlation with the dominant bacteria negatively correlated with SCFAs.

## 4. Discussion

In this study, we analyzed the effects of refined high-fat or low-fat diet on obesity, energy expenditure, gut microbiota, and the metabolites. In the usual stereotype, the HFD was proved to reduce the gut microbiota diversity and production of SCFAs. However, the choice of the control group and differences in nutrient intake are able to alter the usual stereotype. The results of this study revealed that the HFD indeed triggered obesity and disturbed glucose homeostasis. However, the gut microbiota diversity and SCFAs were also increased by the HFD, as well as the energy expenditure. We analyzed the composition of the two diets and dietary intake and found that high-energy-density HFD reduced food intake, but the fiber intake of mice fed HFD was significantly higher than mice fed LFD as the fiber content in HFD was higher than that in LFD, increasing the gut microbiota diversity, metabolites, and energy metabolism.

Mice fed an HFD for 14 weeks had significantly increased body weight, inducing obesity, and disturbed glucose homeostasis, consistent with previous studies [26]. The daily energy intake and intestinal SCFAs in the HFD group were much higher than those in the LFD group, but the mean weight gain was only 4.61 g higher than that of the LFD group. Therefore, we suspected that the mice fed HFD might consume more calories. The metabolic heat production of the HFD group was significantly higher than that of the LFD group, especially in the dark, which was consistent with the living habits of the mice. The RQ of the mice fed HFD was significantly lower than the mice fed LFD, determined by the food composition. Mice in the LFD group mainly used carbohydrates for energy, while the high-fat group metabolized more lipids for energy. Moreover, we tested the expression of related thermogenesis genes *Ucp-1*, *Pgc1α*, *Ppar-α*, and *Ppar-γ* in the BAT, and these genes were significantly up-regulated in the HFD group, consistent with the results of the metabolic rate of heat. Moreover, we detected lipid synthesis and lipolysis related genes in the liver and found that the lipase genes *Atgl* and *Hsl* of the HFD group were significantly up-regulated, and the lipid synthase gene *Fasn* was also significantly up-regulated, indicating that lipid metabolism in mice fed HFD were more active, which also explains why the R.Q. of the HFD group was lower than the LFD group.

Diets provide nutrition to both the host and the gut microbiota. Different diets shape various compositions of the gut microbiome, which consequently affects host metabolism [27,28,29]. Diets can alter host-microbial diversity, and a plant-based diet has been reported as increasing both microbial richness and biodiversity in humans [30]. Matthew et al. reported that both refined HFD and LFD increased the Firmicutes:Bacteroidetes ratio and reduced microbial diversity compared to a standard chow diet in mice, but there was no significant difference in Firmicutes:Bacteroidetes ratio in the ileum, colon, and feces between the mice fed rHFD and LFD [29]. Similarly, in our study, no changes were found in the Firmicutes:Bacteroidetes ratio in the cecum of either the mice fed HFD or LFD. However, HFD increased the alpha diversity in both cecum and colon compared to LFD, and the Firmicutes:Bacteroidetes ratio was significantly decreased in mice fed HFD. These results are quite different from those reported by previous studies and probably due to the different choices of control diets. If the standard chow diet is chosen as the control group, the results usually show that the HFD decreases the abundance of gut bacteria, increases the Firmicutes:Bacteroidetes ratio, and decreases SCFAs, because the standard chow diet is cereal-based, and it contains much more fiber (about 150 g/kg) than rHFD or rLFD. Furthermore, it has not been found that high fat or high protein can increase the diversity of gut microbiota and SCFAs, but non-absorbed carbohydrates (e.g., inulin, cellulose) are able to achieve these results [30]. In our study, the fiber intake of mice fed HFD was significantly higher than that of mice fed LFD. Therefore, it is believed that the higher content of fiber in HFD may be the cause of increased gut microbiota diversity and SCFAs in the HFD group.

In the HFD group, the most abundant bacterium in the cecum and the colon was an uncultured Desulfovibrionaceae bacterium and an uncultured Muribaculaceae bacterium, respectively. In contrast, in the LFD group, *Lactobacillus* was the most abundant genus in both the cecum and colon. Desulfovibrionaceae has been proved to up-regulate CD36 expression and increase uptake of long-chain fatty acids, leading to metabolic disease [31]. The HFD altered the gut ecosystem and drove colonization by bacteria that can utilize the abundant nutrients (e.g., fatty acids). Therefore, large numbers of Desulfovibrionaceae colonized the cecum of the HFD group mice. Meanwhile, the relative abundance of *Lactobacillus* in the cecum and colon was much lower in the HFD group, in agreement with previous reports [32,33,34].

The *Rikenellaceae RC9 gut group* and *Mucispirillum* were identified by VIP predictive and LEfSe as having a higher relative abundance in the HFD group. Previous studies have reported that HFD enriched the abundance of both the *Rikenellaceae RC9 gut group* and *Mucispirillum* in the cecum [35,36], with *Mucispirillum* being correlated positively with the presence of non-alcoholic steatohepatitis. Compared with cecal microbiota, there were more OTUs observed in the colonic microbiota. The abundances of Muribaculaceae, *Rikenellaceae RC9 gut group*, *Odoribacter*, *Mucispirillum*, *Alistipes*, uncultured Muribaculaceae bacteria (two OTUs), and an uncultured Bacteroidales bacterium were higher in the HFD group, while *Faecalibaculum*, *Blautia*, *Bifidobacterium*, *Akkermansia*, and an uncultured Muribaculaceae bacterium were identified as having a lower abundance in the HFD group.

Muribaculaceae are also named family S24-7, which is a dominant family in the gut microbiome [37,38], and produce enzymes that degrade complex carbohydrates. Previous studies have identified Muribaculaceae as capable of degrading carbohydrates so that high-calorie diets decreased the abundance of the bacteria [39,40]. However, Cao et al. noted that Muribaculaceae in obesity-resistant mice was more abundant than that in obese mice, despite being fed the same HFD [41]. In this study, Muribaculaceae was more abundant in the HFD group than the LFD group, both in the cecum and the colon. As the fiber intake of mice fed HFD was significantly higher than mice fed LFD, we concluded that dietary fiber drove the different abundance of Muribaculaceae in two groups. Due to the limitations of current sequencing methods, we could not determine whether specific members of Muribaculaceae promoted obesity, but Muribaculaceae undoubtedly plays a vital role in modulating energy metabolism in mice. HFD significantly increased the abundance of Rikenellaceae [38,42], including the genus *Alistipes*, which was thought to be related to type 2 diabetes mellitus (T2DM) [43], consistent with our results. *Mucispirillum* is a pathobiont widely distributed throughout the digestive tract, with flagella that allow it to cross the mucosal barrier [43], which may induce intestinal inflammation and lead to colitis [44,45,46]. In this study, HFD increased the genus *Mucispirillum* in the cecum and colon, which aggravated the risk of host inflammation. The abundances of probiotics such as *Lactobacillus* and *Bifidobacterium* were decreased by the HFD, consistent with previous studies [47,48], as well as potential probiotics such as *Akkermansia* [16], *Faecalibaculum* [37], and *Blautia* [49]. It has been reported that these bacteria alleviate metabolic diseases or that their presence is inversely related to the development of metabolic diseases. Although increased fiber intake alleviated gut microbiota diversity, it did not reverse the adverse effects of high fat on the colonization of beneficial bacteria. On the one hand, the fiber intake in this study is far lower than the amount of fiber intervention thought to be needed (15–25% fiber). On the other hand, part of the significant impacts on gut microbiota by high fat is not so easy to reverse.

Besides modeling the gut microbiota, diets altered the production of metabolites such as SCFAs. The refined LFD contained a large proportion of carbohydrates, but lower fiber. The total concentration of SCFAs was lower in the LFD group compared to the HFD group. Studies show that a high-fiber diet increases gut microbial richness and biodiversity, as well as the production of SCFAs, compared to a Western diet [30]. Matthew et al. revealed that a standard chow diet containing threefold more fiber significantly increased the production of SCFAs in mice compared to mice fed a refined HFD or LFD [29]. The article showed that both the refined HFD (D12492) and LFD (D12450J) contained 50 g/kg fiber. The fiber content was 50.00 g/773.85 g in the D12492 and 50 g/1055.05 g in the D12450J. However, the actual value of fiber content was 64.61 g/kg in the D12492 and 47.39 g/kg in the D12450J. The fiber intake in the refined HFD was significantly higher than that in the refined LFD in that study. Although there was no difference in the cecal SCFAs between the refined HFD and LFD, the HFD increased the Shannon diversity index of the gut microbiota [29]. In the animal studies of the effect of fiber on obesity, the diets contained 15–25% fiber, and the fiber significantly improved the metabolic status. Interestingly, in this study, the fiber intake from HFD was much lower than the fiber intervention of previous studies, but it significantly affected the energy metabolism, gut microbiota, and its metabolites.

The predicted functions of the gut microbiota showed that the gut microbiota induced by the HFD might have stronger drug resistance and increase the risk of colonization of pathogenic bacteria and inflammation than that induced by the LFD. These results were consistent with the results of the gut microbiota analysis above. The predicted functional compositions of pyruvate metabolism, propanoate metabolism, and butanoate metabolism pathways were significantly up-regulated by the HFD. These pathways are the main pathways for non-absorbed carbohydrate metabolism, and their metabolites are SCFAs. Fiber cannot be degraded in the small intestine, and it is mainly fermented by the gut microbiota in the large intestine to produce SCFAs. The increased dietary fiber from the HFD increased the production of SCFAs in the large intestine, which is consistent with predicted functional compositions of pathways for the metabolism of non-absorbed carbohydrates.

Studies have shown that SCFAs are beneficial for energy metabolism, promoting intestinal gluconeogenesis, and increasing energy consumption [50,51,52]. The predicted functional compositions of the glycolysis/gluconeogenesis pathway were significantly up-regulated in the HFD group, causing increased SCFAs. Butyric acid has been proved to up-regulate the thermogenesis-related genes *Pgc1α* and *Ucp-1* in the BAT and increase energy expenditure [50,51]. In this study, we found that the amount of butyric acid in the cecum was significantly higher in the HFD group than in the LFD group, which probably caused the up-regulation of thermogenesis gene expression in BAT and increased energy expenditure. The energy density is different between the HFD and LFD, influencing the specific nutrient (e.g., fiber) intake in mice. It is necessary to evaluate whether the differences in specific nutrient intake will interfere with the results of animal experiments. The choice and optimization of the dietary formula are indispensable for studies on metabolic diseases. Besides, there are limitations in this study, a high-fiber and high-fat diet group will be employed in further experiments. We will conduct more focus on the impact of different amounts of fiber intake on host metabolic health.

## 5. Conclusions

The increased fiber intake from the high-fat diet increased the gut microbiota diversity and the production of SCFAs in the large intestine, thereby increasing the metabolic rates and thermogenesis in the BAT. As it lacked dietary fiber, the LFD reduced the diversity of gut microbiota or the production of SCFAs; however, it had beneficial effects on glucose control and promoted gut colonization by beneficial bacteria. The high-fat diet was rich in lipids and proteins, which increased the risks of colonization of intestinal pathogens and inflammation. In this study, the difference in fiber intake significantly altered energy metabolism, gut microbiota, and its metabolites. It is necessary for researchers to evaluate the effect of dietary formula in studies and whether differences in specific nutrient intake will interfere with the results of animal experiments. Moreover, humans should explore lowering the proportion of dietary fat consumed and increasing fiber intake to raise gut microbiota diversity and thus promote gut health.

## Figures and Tables

**Table 1:** The diet composition of the high-fat diet (TP 23300) and low-fat, high-carbohydrate diet (TP 23302) ^1^.

Ingredient, g/kg	TP 23300	TP 23302
Casein	259	189
Maltodextrin	166	108
Sucrose	91	67
Corn Starch	0	497
Soybean Oil	33	21
Lard	313	21
Cellulose	67	48
Mineral Mix, M1020	52	36
Vitamin Mix, V1010	13	9
L-Cystine	3	2
Choline Bitartrate	3	2
Tertiary butylhydroquinone (TBHQ)	0.067	0.067
Total	1000.067	1000.067

^1^ The purified diet TP 23,300 supplies energy as 20% carbohydrate (7% sucrose calories), 20% protein, 60% fat with total 5.1 kcal/g; and the purified diet TP23302 supplies energy as 70% carbohydrate (7% sucrose calories), 20% protein, 10% fat with total 3.8 kcal/g.

**Table 2:** Primers for real-time polymerase chain reaction (PCR) analysis of gene expression ^1^.

Gene	PrimerBank ID	Forward Primer	Reverse Primer
*Ucp1*	6678497a1	AGGCTTCCAGTACCATTAGGT	CTGAGTGAGGCAAAGCTGATTT
*Pgc1α*	6679433a1	TATGGAGTGACATAGAGTGTGCT	CCACTTCAATCCACCCAGAAAG
*Ppar-α*	31543500a1	AGAGCCCCATCTGTCCTCTC	ACTGGTAGTCTGCAAAACCAAA
*Ppar-γ*	6755138a1	TCGCTGATGCACTGCCTATG	GAGAGGTCCACAGAGCTGATT
*Fasn*	30911099a1	GGAGGTGGTGATAGCCGGTAT	TGGGTAATCCATAGAGCCCAG
*Atgl*	26327465a1	GGATGGCGGCATTTCAGACA	CAAAGGGTTGGGTTGGTTCAG
*Mgl*	6754690a1	CGGACTTCCAAGTTTTTGTCAGA	GCAGCCACTAGGATGGAGATG
*Hsl*	26325924a1	CCAGCCTGAGGGCTTACTG	CTCCATTGACTGTGACATCTCG
*Gapdh*	6679937a1	AGGTCGGTGTGAACGGATTTG	TGTAGACCATGTAGTTGAGGTCA

^1^ The validated primers were obtained from Primerbank.
